# Crystal structure of tetra­aqua­bis(3,5-di­amino-4*H*-1,2,4-triazol-1-ium)cobalt(II) bis­[bis­(pyridine-2,6-di­carboxyl­ato)cobaltate(II)] dihydrate

**DOI:** 10.1107/S2056989015010014

**Published:** 2015-05-30

**Authors:** Atim Johnson, Justina Mbonu, Zahid Hussain, Wan-Sin Loh, Hoong-Kun Fun

**Affiliations:** a H. E. J. Research Institute of Chemistry, International Center for Chemical and Biological Sciences, University of Karachi, Karachi 75720, Pakistan; b Department of Chemistry, University of Uyo, P.M.B. 1017, Uyo, Akwa Ibom State, Nigeria; c Department of Chemistry, Federal University of Petroleum Recourses Effurun, Delta State, Nigeria; d Department of Chemistry, Karakoram International University, Gilgit, Baltistan, Pakistan; e X-ray Crystallography Unit, School of Physics, Universiti Sains Malaysia, 11800 USM, Penang, Malaysia; f Department of Pharmaceutical Chemistry, College of Pharmacy, King Saud University, PO Box 2457, Riaydh 11451, Saudi Arabia

**Keywords:** crystal structure, pyridine-2,6-di­carboxyl­ate, triazolium, Co^II^ complex, hydrogen bonding

## Abstract

The asymmetric unit of the title compound, [Co(C_2_H_6_N_5_)_2_(H_2_O)_4_][Co(C_7_H_3_NO_4_)_2_]_2_·2H_2_O, features 1.5 Co^II^ ions (one anionic complex and one half cationic complex) and one water mol­ecule. In the cationic complex, the Co^II^ atom is located on an inversion centre and is coordinated by two triazolium cations and four water mol­ecules, adopting an octa­hedral geometry where the N atoms of the two triazolium cations occupy the axial positions and the O atoms of the four water mol­ecules the equatorial positions. The two triazole ligands are parallel offset (with a distance of 1.38 Å between their planes). In the anionic complex, the Co^II^ ion is six-coordinated by two N and four O atoms of the two pyridine-2,6-di­carboxyl­ate anions, exhibiting a slightly distorted octa­hedral coordination geometry in which the mean plane of the two pyridine-2,6-di­carboxyl­ate anions are almost perpendicular to each other, making a dihedral angle of 85.87 (2)°. In the crystal, mol­ecules are linked into a three-dimensional network *via* C—H⋯O, C—H⋯N, O—H⋯O and N—H⋯O hydrogen bonds.

## Related literature   

For the different coordination modes of transition metal–dipicolinate complexes, see: Håkansson *et al.* (1993 ▸); Okabe & Oya (2000 ▸); Aghajani *et al.* (2009 ▸). For crystal structures of related complexes, see: Yousuf *et al.* (2011*a* ▸,*b* ▸); Aghabozorg *et al.* (2009 ▸); Ramos Silva *et al.* (2008 ▸); Wang *et al.* (2004 ▸); MacDonald *et al.* (2004 ▸). For studies on proton transfer from carb­oxy­lic acids to both heterocyclic and substituted amine N atoms, see: Aghabozorg *et al.* (2008 ▸); Moghimi *et al.* (2002 ▸, 2005 ▸, 2007 ▸); Pasdar *et al.* (2011 ▸); Tabatabaee *et al.* (2009 ▸).
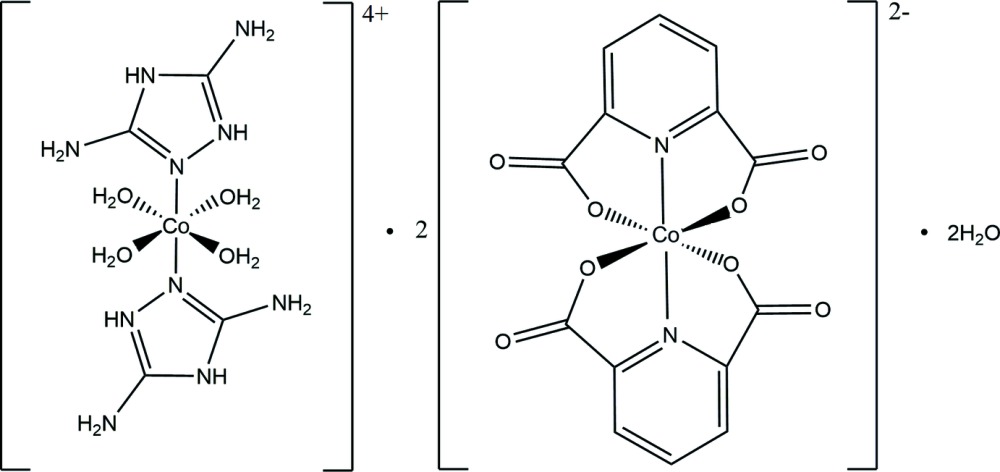


## Experimental   

### Crystal data  

[Co(C_2_H_6_N_5_)_2_(H_2_O)_4_][Co(C_7_H_3_NO_4_)_2_]_2_·2H_2_O
*M* *_r_* = 1145.54
Monoclinic, 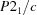

*a* = 7.1499 (2) Å
*b* = 10.8807 (2) Å
*c* = 26.6877 (6) Å
β = 90.649 (1)°
*V* = 2076.06 (8) Å^3^
*Z* = 2
Mo *K*α radiation
μ = 1.29 mm^−1^
*T* = 100 K
0.43 × 0.28 × 0.28 mm

### Data collection  

Bruker SMART APEXII CCD area-detector diffractometer
Absorption correction: multi-scan (*SADABS*; Bruker, 2009 ▸) *T*_min_ = 0.607, *T*_max_ = 0.718
35534 measured reflections
9081 independent reflections
8203 reflections with *I* > 2σ(*I*)
*R*_int_ = 0.021

### Refinement  

*R*[*F* ^2^ > 2σ(*F* ^2^)] = 0.024
*wR*(*F* ^2^) = 0.068
*S* = 1.05
9081 reflections
370 parameters
H atoms treated by a mixture of independent and constrained refinement
Δρ_max_ = 0.56 e Å^−3^
Δρ_min_ = −0.31 e Å^−3^

### 

Data collection: *APEX2* (Bruker, 2009 ▸); cell refinement: *SAINT* (Bruker, 2009 ▸); data reduction: *SAINT*; program(s) used to solve structure: *SHELXS97* (Sheldrick 2008 ▸); program(s) used to refine structure: *SHELXL2013* (Sheldrick, 2015 ▸); molecular graphics: *SHELXTL* (Sheldrick 2008 ▸); software used to prepare material for publication: *SHELXTL* and *PLATON* (Spek, 2009 ▸).

## Supplementary Material

Crystal structure: contains datablock(s) I, global. DOI: 10.1107/S2056989015010014/zl2619sup1.cif

**Fig. 1.**
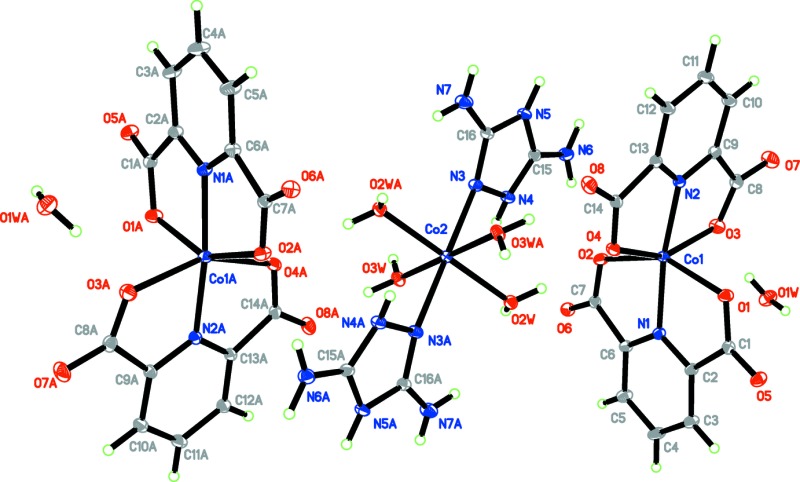
The molecular structure of the title compound with atom labels and 50% probability displacement ellipsoids. Atoms with suffix A were generated by the symmetry operation -x, -y, -z.

Structure factors: contains datablock(s) I. DOI: 10.1107/S2056989015010014/zl2619Isup2.hkl

x y z . DOI: 10.1107/S2056989015010014/zl2619fig1.tif
The mol­ecular structure of the title compound with atom labels and 50% probability displacement ellipsoids. Atoms with suffix A were generated by the symmetry operation −*x*, −*y*, −*z*.

**Fig. 2.**
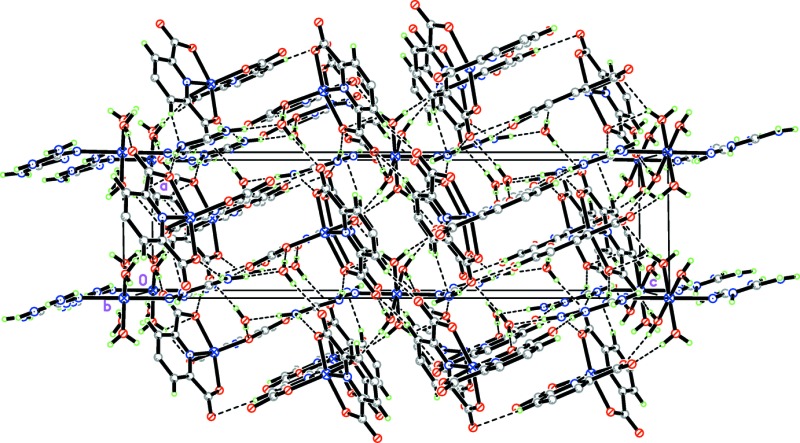
Crystal packing of the title compound, showing the three-dimensional network. H atoms not involved in the intermolecular interactions (dashed lines) have been omitted for clarity.

. DOI: 10.1107/S2056989015010014/zl2619fig2.tif
Crystal packing of the title compound, showing the three-dimensional network. H atoms not involved in the inter­molecular inter­actions (dashed lines) have been omitted for clarity.

CCDC reference: 1402526

Additional supporting information:  crystallographic information; 3D view; checkCIF report

## Figures and Tables

**Table 1:** Hydrogen-bond geometry (, )

*D*H*A*	*D*H	H*A*	*D* *A*	*D*H*A*
N4H1*N*4O1^i^	0.88(2)	1.74(2)	2.6044(12)	165(2)
N5H1*N*5O7^ii^	0.89(2)	1.77(2)	2.6402(12)	169(2)
N6H1*N*6O2	0.84(2)	2.15(2)	2.9117(12)	151.1(19)
N6H2*N*6O1*W* ^ii^	0.92(2)	1.85(2)	2.7645(12)	173.6(19)
N7H2*N*7O6^iii^	0.80(2)	2.24(2)	2.9956(13)	158.4(18)
O1*W*H1*W*1O3	0.83(2)	1.90(2)	2.7354(13)	171(3)
O1*W*H2*W*1O6^iv^	0.79(2)	2.09(2)	2.8711(12)	175(2)
O2*W*H1*W*2O5^i^	0.86(2)	1.80(2)	2.6666(11)	173(2)
O2*W*H2*W*2O4	0.82(2)	2.00(2)	2.8206(11)	175(2)
O3*W*H1*W*3O4^i^	0.79(2)	2.19(2)	2.9602(11)	163(2)
O3*W*H2*W*3O8^v^	0.83(2)	1.76(2)	2.5795(11)	173(2)
C3H3*A*O5^vi^	0.95	2.25	3.1816(13)	167
C5H5*A*N7^vii^	0.95	2.50	3.4265(15)	164
C10H10*A*O6^ii^	0.95	2.44	3.3898(13)	177

Symmetry codes: (i) 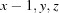; (ii) 
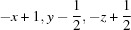
; (iii) 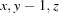; (iv) 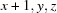; (v) 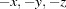; (vi) 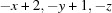; (vii) 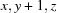.

## supplementary crystallographic information

### S1. Chemical context

Among different multidentate species, compounds bearing carboxyl­ate functions are widely studied ligands for producing stable transition metals coordination polymers and supra­molecular architectures, mostly because of the versatile ligating abilities of the -COO moieties and also due to the enhanced affinity of these metal ions towards such O donors. There have been a number of successful attempts at utilizing proton transfer from carb­oxy­lic acids to both heterocyclic and substituted amine nitro­gens (Moghimi *et al.*, 2002; Moghimi *et al.*, 2005; Moghimi *et al.*, 2007; Aghabozorg *et al.*, 2008; Aghabozorg *et al.*, 2009; Tabatabaee *et al.*, 2009, Pasdar *et al.*, 2011, Yousuf *et al.*, 2011*b*). Di­carb­oxy­lic acids possess a good potential to be used as proton donors in the synthesis of proton transfer compounds. In continuation of our work, we report herein the trinuclear complex of Co^II^ with pyridine-2,6-di­carb­oxy­lic acid as proton donor and 3,5-di­amino-1,2,4-triazole as proton acceptor.

### S2. Structural commentary

The asymmetric unit consits of half whole repeating unit of the title compound (Fig. 1) and is composed of 1.5 Co^II^ ions (one anionic complex and one half cationic complex) and one water molecule. In the cationic complex, the Co^II^ atom (Co2) is located on an inversion centre and is coordinated by two triazolium cations and four water molecules, adopting an o­cta­hedral geometry where the N atoms of the two triazolium cations (N3 & N3A) occupy the axial positions (Co–N = 2.2016 (7) Å) and the O atoms of the four water molecules (O2W, O2WA, O3W & O3WA) occupy the equatorial positions (Co–O = 2.0590 (7) - 2.1080 (7) Å). Atoms with suffix A were generated by the symmetry operation -*x*, -*y*, -*z*. The two triazole ligands are parallel offset (with a distance of 1.377 Å between the exact parallel planes). The angle between the Co–N bond and the centroid of the triazole plane is 158.56°. The bond distances are comparable with those reported for similar complexes (Aghabozorg *et al.*, 2008; Prasad & Rajasekharan, 2007; Colak *et al.*, 2009). In the anionic complex, the Co^II^ ion (Co1) is six-coordinated by two N (Co–N = 2.0273 (9) - 2.0308 (9) Å) and four O (Co–O = 2.1471 (7) - 2.2223 (7) Å) atoms of the two pyridine-2,6-di­carboxyl­ate anions, exhibiting a slightly distorted o­cta­hedral coordination geometry where the mean plane of the two pyridine-2,6-di­carboxyl­ate anions (maximum deviation = 0.0851 (9) Å at C7) are almost perpendicular to each other with a dihedral angle of 85.87 (2)°.

### S3. Supra­molecular features

In the crystal packing (Fig. 2), the molecules are linked into a three dimensional network *via* inter­molecular C—H···O, C—H···N, O—H···O and N—H···O hydrogen bonds (Table 1).

### S4. Synthesis and crystallization

An aqueous solution (10 ml) containing 0.5mmol (0.0496 g) of 3,5-di­amino-1,2,4-triazole was added to a hot and stirring aqueous solution (20 ml) containing 1mmol (0.167 g) of pyridine-2,6-di­carb­oxy­lic acid and 1mmol (0.238 g) of CoCl_2_.6H_2_O. The resulting pink solution was stirred for 30 min and allowed to stand at room temperature. Crystals formed after 3 days but single crystals suitable for X-ray analysis were separated after one month.

### S5. Refinement details

N- and O- bound H atoms were located from the difference Fourier map and were refined freely [N—H = 0.79 (2) to 0.92 (2) Å; O—H = 0.79 (2) to 0.86 (2) Å]. The remaining H atoms were calculated geometrically and were refined using a riding model with U_iso_ = 1.2 *U*_eq_(C), with the bond lengths of C–H being 0.95 Å.

### Crystal data

**Table:**

[Co(C_2_H_6_N_5_)_2_(H_2_O)_4_][Co(C_7_H_3_NO_4_)_2_]_2_·2H_2_O	*F*(000) = 1166
*M**_r_* = 1145.54	*D*_x_ = 1.833 Mg m^−^^3^
Monoclinic, *P*2_1_/*c*	Mo *K*α radiation, λ = 0.71073 Å
*a* = 7.1499 (2) Å	Cell parameters from 9904 reflections
*b* = 10.8807 (2) Å	θ = 2.4–35.0°
*c* = 26.6877 (6) Å	µ = 1.29 mm^−^^1^
β = 90.649 (1)°	*T* = 100 K
*V* = 2076.06 (8) Å^3^	Block, purple
*Z* = 2	0.43 × 0.28 × 0.28 mm

### Data collection

**Table:**

Bruker SMART APEXII CCD area-detector diffractometer	9081 independent reflections
Radiation source: fine-focus sealed tube	8203 reflections with *I* > 2σ(*I*)
Graphite monochromator	*R*_int_ = 0.021
φ and ω scans	θ_max_ = 35.1°, θ_min_ = 1.5°
Absorption correction: multi-scan (*SADABS*; Bruker, 2009)	*h* = −9→11
*T*_min_ = 0.607, *T*_max_ = 0.718	*k* = −17→17
35534 measured reflections	*l* = −42→41

### Refinement

**Table:**

Refinement on *F*^2^	0 restraints
Least-squares matrix: full	Hydrogen site location: mixed
*R*[*F*^2^ > 2σ(*F*^2^)] = 0.024	H atoms treated by a mixture of independent and constrained refinement
*wR*(*F*^2^) = 0.068	*w* = 1/[σ^2^(*F*_o_^2^) + (0.0314*P*)^2^ + 1.0292*P*] where *P* = (*F*_o_^2^ + 2*F*_c_^2^)/3
*S* = 1.05	(Δ/σ)_max_ = 0.001
9081 reflections	Δρ_max_ = 0.56 e Å^−^^3^
370 parameters	Δρ_min_ = −0.31 e Å^−^^3^

### Special details

**Table:**

Geometry. All e.s.d.'s (except the e.s.d. in the dihedral angle between two l.s. planes) are estimated using the full covariance matrix. The cell e.s.d.'s are taken into account individually in the estimation of e.s.d.'s in distances, angles and torsion angles; correlations between e.s.d.'s in cell parameters are only used when they are defined by crystal symmetry. An approximate (isotropic) treatment of cell e.s.d.'s is used for estimating e.s.d.'s involving l.s. planes.

### Fractional atomic coordinates and isotropic or equivalent isotropic displacement parameters (Å^2^)

**Table:**

	*x*	*y*	*z*	*U*_iso_*/*U*_eq_	
Co1	0.55162 (2)	0.25891 (2)	0.13399 (2)	0.00894 (3)	
Co2	0.0000	0.0000	0.0000	0.00821 (4)	
O1	0.82104 (10)	0.26153 (6)	0.09788 (3)	0.01282 (12)	
O2	0.27767 (10)	0.33471 (6)	0.14695 (3)	0.01253 (12)	
O3	0.65732 (11)	0.31798 (7)	0.20566 (3)	0.01412 (12)	
O4	0.46908 (10)	0.11281 (6)	0.07992 (2)	0.01164 (11)	
O5	0.98594 (10)	0.35876 (7)	0.03895 (3)	0.01370 (12)	
O6	0.11089 (10)	0.50933 (6)	0.13722 (3)	0.01255 (12)	
O7	0.79218 (12)	0.24719 (7)	0.27668 (3)	0.01834 (14)	
O8	0.44182 (11)	−0.09334 (7)	0.07506 (3)	0.01482 (13)	
N1	0.54987 (11)	0.41617 (7)	0.09324 (3)	0.00998 (12)	
N2	0.59941 (11)	0.09926 (7)	0.17147 (3)	0.00943 (12)	
N3	−0.00429 (11)	−0.02963 (7)	0.08158 (3)	0.01012 (12)	
N4	0.03927 (11)	0.07247 (7)	0.11156 (3)	0.01056 (12)	
N5	0.08642 (11)	−0.08746 (7)	0.15824 (3)	0.01057 (12)	
N6	0.13643 (13)	0.11292 (8)	0.19445 (3)	0.01373 (14)	
N7	−0.00180 (14)	−0.24234 (8)	0.09908 (3)	0.01487 (15)	
C1	0.85009 (12)	0.34939 (8)	0.06704 (3)	0.01023 (14)	
C2	0.69856 (12)	0.44549 (8)	0.06584 (3)	0.01026 (14)	
C3	0.70679 (14)	0.55422 (9)	0.03875 (4)	0.01333 (15)	
H3A	0.8119	0.5733	0.0187	0.016*	
C4	0.55548 (15)	0.63436 (9)	0.04196 (4)	0.01642 (17)	
H4A	0.5576	0.7105	0.0246	0.020*	
C5	0.40079 (14)	0.60293 (9)	0.07070 (4)	0.01499 (16)	
H5A	0.2967	0.6568	0.0730	0.018*	
C6	0.40236 (13)	0.49099 (8)	0.09586 (3)	0.01047 (14)	
C7	0.24820 (12)	0.44216 (8)	0.12908 (3)	0.00996 (13)	
C8	0.71223 (13)	0.23375 (9)	0.23540 (3)	0.01208 (15)	
C9	0.67492 (13)	0.10389 (8)	0.21746 (3)	0.01040 (14)	
C10	0.71742 (14)	−0.00249 (9)	0.24395 (3)	0.01303 (15)	
H10A	0.7708	0.0016	0.2767	0.016*	
C11	0.67989 (14)	−0.11541 (9)	0.22139 (4)	0.01395 (15)	
H11A	0.7070	−0.1896	0.2388	0.017*	
C12	0.60241 (13)	−0.11954 (8)	0.17318 (3)	0.01207 (15)	
H12A	0.5762	−0.1957	0.1572	0.014*	
C13	0.56501 (12)	−0.00872 (8)	0.14938 (3)	0.00955 (13)	
C14	0.48503 (12)	0.00302 (8)	0.09707 (3)	0.00989 (14)	
C15	0.09005 (12)	0.03737 (8)	0.15732 (3)	0.01009 (14)	
C16	0.02562 (12)	−0.12445 (8)	0.11143 (3)	0.00985 (13)	
O1W	0.81160 (12)	0.54803 (8)	0.20623 (3)	0.01822 (14)	
O2W	0.16935 (10)	0.15580 (6)	0.01259 (3)	0.01110 (11)	
O3W	−0.23235 (10)	0.11122 (7)	0.00395 (3)	0.01179 (11)	
H1N4	−0.017 (2)	0.1420 (17)	0.1043 (6)	0.024 (4)*	
H1N5	0.124 (3)	−0.1360 (19)	0.1831 (7)	0.037 (5)*	
H1N6	0.147 (2)	0.1885 (17)	0.1873 (7)	0.026 (4)*	
H2N6	0.151 (3)	0.0852 (17)	0.2268 (7)	0.030 (5)*	
H1N7	−0.047 (3)	−0.2572 (17)	0.0707 (8)	0.030 (5)*	
H2N7	0.024 (2)	−0.2988 (17)	0.1167 (6)	0.023 (4)*	
H1W1	0.753 (3)	0.482 (2)	0.2054 (8)	0.043 (6)*	
H2W1	0.899 (3)	0.5365 (19)	0.1887 (8)	0.038 (5)*	
H1W2	0.113 (3)	0.2197 (18)	0.0235 (7)	0.030 (5)*	
H2W2	0.261 (3)	0.1440 (18)	0.0306 (7)	0.032 (5)*	
H1W3	−0.295 (3)	0.1150 (18)	0.0282 (7)	0.035 (5)*	
H2W3	−0.308 (3)	0.1054 (18)	−0.0198 (7)	0.033 (5)*	

### Atomic displacement parameters (Å^2^)

**Table:**

	*U*^11^	*U*^22^	*U*^33^	*U*^12^	*U*^13^	*U*^23^
Co1	0.01044 (5)	0.00761 (5)	0.00876 (5)	0.00044 (4)	0.00033 (4)	0.00075 (4)
Co2	0.00864 (7)	0.00831 (7)	0.00767 (7)	0.00067 (5)	−0.00032 (5)	0.00030 (5)
O1	0.0124 (3)	0.0111 (3)	0.0150 (3)	0.0025 (2)	0.0028 (2)	0.0036 (2)
O2	0.0126 (3)	0.0105 (3)	0.0145 (3)	0.0007 (2)	0.0027 (2)	0.0029 (2)
O3	0.0188 (3)	0.0109 (3)	0.0126 (3)	−0.0005 (2)	−0.0018 (2)	−0.0012 (2)
O4	0.0143 (3)	0.0100 (3)	0.0106 (3)	0.0002 (2)	−0.0021 (2)	0.0011 (2)
O5	0.0120 (3)	0.0122 (3)	0.0170 (3)	0.0011 (2)	0.0051 (2)	0.0012 (2)
O6	0.0118 (3)	0.0128 (3)	0.0132 (3)	0.0027 (2)	0.0025 (2)	0.0001 (2)
O7	0.0262 (4)	0.0160 (3)	0.0126 (3)	−0.0015 (3)	−0.0069 (3)	−0.0036 (2)
O8	0.0184 (3)	0.0113 (3)	0.0146 (3)	0.0006 (2)	−0.0057 (2)	−0.0035 (2)
N1	0.0101 (3)	0.0093 (3)	0.0105 (3)	0.0005 (2)	0.0017 (2)	0.0006 (2)
N2	0.0102 (3)	0.0097 (3)	0.0084 (3)	−0.0006 (2)	−0.0005 (2)	−0.0002 (2)
N3	0.0127 (3)	0.0083 (3)	0.0093 (3)	0.0014 (2)	−0.0004 (2)	0.0004 (2)
N4	0.0143 (3)	0.0086 (3)	0.0088 (3)	0.0013 (2)	−0.0007 (2)	0.0005 (2)
N5	0.0129 (3)	0.0102 (3)	0.0086 (3)	0.0006 (2)	−0.0010 (2)	0.0019 (2)
N6	0.0190 (4)	0.0125 (3)	0.0097 (3)	−0.0029 (3)	−0.0009 (3)	−0.0002 (2)
N7	0.0210 (4)	0.0088 (3)	0.0147 (3)	0.0004 (3)	−0.0025 (3)	0.0008 (3)
C1	0.0099 (3)	0.0093 (3)	0.0116 (3)	0.0001 (3)	0.0004 (3)	−0.0006 (3)
C2	0.0103 (3)	0.0090 (3)	0.0115 (3)	0.0007 (3)	0.0017 (3)	0.0006 (3)
C3	0.0135 (4)	0.0095 (3)	0.0170 (4)	0.0003 (3)	0.0046 (3)	0.0027 (3)
C4	0.0166 (4)	0.0112 (4)	0.0216 (4)	0.0031 (3)	0.0070 (3)	0.0059 (3)
C5	0.0146 (4)	0.0107 (4)	0.0198 (4)	0.0032 (3)	0.0058 (3)	0.0040 (3)
C6	0.0107 (3)	0.0097 (3)	0.0111 (3)	0.0014 (3)	0.0018 (3)	0.0006 (3)
C7	0.0104 (3)	0.0104 (3)	0.0091 (3)	−0.0003 (3)	0.0007 (3)	−0.0002 (3)
C8	0.0133 (4)	0.0122 (4)	0.0108 (3)	−0.0013 (3)	0.0000 (3)	−0.0027 (3)
C9	0.0116 (3)	0.0115 (3)	0.0081 (3)	−0.0011 (3)	−0.0007 (3)	−0.0006 (3)
C10	0.0155 (4)	0.0137 (4)	0.0099 (3)	−0.0011 (3)	−0.0022 (3)	0.0015 (3)
C11	0.0172 (4)	0.0117 (4)	0.0129 (4)	−0.0004 (3)	−0.0024 (3)	0.0037 (3)
C12	0.0143 (4)	0.0094 (3)	0.0125 (3)	−0.0012 (3)	−0.0022 (3)	0.0011 (3)
C13	0.0101 (3)	0.0094 (3)	0.0091 (3)	−0.0010 (3)	−0.0009 (3)	0.0000 (3)
C14	0.0097 (3)	0.0100 (3)	0.0100 (3)	0.0002 (3)	−0.0008 (3)	−0.0006 (3)
C15	0.0103 (3)	0.0110 (3)	0.0090 (3)	0.0005 (3)	0.0008 (3)	0.0012 (3)
C16	0.0101 (3)	0.0098 (3)	0.0097 (3)	0.0010 (3)	0.0006 (3)	0.0010 (3)
O1W	0.0221 (4)	0.0180 (3)	0.0146 (3)	−0.0055 (3)	0.0041 (3)	−0.0028 (3)
O2W	0.0109 (3)	0.0105 (3)	0.0118 (3)	0.0005 (2)	−0.0007 (2)	0.0000 (2)
O3W	0.0110 (3)	0.0155 (3)	0.0088 (3)	0.0026 (2)	−0.0005 (2)	−0.0011 (2)

### Geometric parameters (Å, º)

**Table:**

Co1—N1	2.0275 (8)	N6—C15	1.3269 (12)
Co1—N2	2.0315 (8)	N6—H1N6	0.848 (19)
Co1—O3	2.1471 (7)	N6—H2N6	0.921 (18)
Co1—O2	2.1567 (7)	N7—C16	1.3383 (12)
Co1—O1	2.1640 (7)	N7—H1N7	0.84 (2)
Co1—O4	2.2223 (7)	N7—H2N7	0.795 (18)
Co2—O3W	2.0590 (7)	C1—C2	1.5058 (13)
Co2—O3W^i^	2.0590 (7)	C2—C3	1.3880 (13)
Co2—O2W^i^	2.1080 (7)	C3—C4	1.3929 (14)
Co2—O2W	2.1080 (7)	C3—H3A	0.9500
Co2—N3^i^	2.2015 (7)	C4—C5	1.3957 (13)
Co2—N3	2.2016 (7)	C4—H4A	0.9500
O1—C1	1.2800 (11)	C5—C6	1.3908 (13)
O2—C7	1.2792 (11)	C5—H5A	0.9500
O3—C8	1.2715 (12)	C6—C7	1.5183 (12)
O4—C14	1.2840 (11)	C8—C9	1.5146 (13)
O5—C1	1.2378 (11)	C9—C10	1.3881 (13)
O6—C7	1.2451 (11)	C10—C11	1.3928 (14)
O7—C8	1.2440 (11)	C10—H10A	0.9500
O8—C14	1.2392 (11)	C11—C12	1.3959 (13)
N1—C6	1.3347 (12)	C11—H11A	0.9500
N1—C2	1.3358 (11)	C12—C13	1.3873 (12)
N2—C13	1.3361 (11)	C12—H12A	0.9500
N2—C9	1.3363 (11)	C13—C14	1.5080 (12)
N3—C16	1.3194 (11)	O1W—H1W1	0.83 (2)
N3—N4	1.4020 (11)	O1W—H2W1	0.79 (2)
N4—C15	1.3261 (11)	O2W—H1W2	0.86 (2)
N4—H1N4	0.877 (18)	O2W—H2W2	0.817 (19)
N5—C15	1.3587 (12)	O3W—H1W3	0.79 (2)
N5—C16	1.3781 (12)	O3W—H2W3	0.830 (19)
N5—H1N5	0.89 (2)		
			
N1—Co1—N2	170.32 (3)	O5—C1—O1	125.80 (9)
N1—Co1—O3	103.02 (3)	O5—C1—C2	119.95 (8)
N2—Co1—O3	76.22 (3)	O1—C1—C2	114.23 (8)
N1—Co1—O2	76.30 (3)	N1—C2—C3	121.83 (8)
N2—Co1—O2	113.33 (3)	N1—C2—C1	113.54 (8)
O3—Co1—O2	93.09 (3)	C3—C2—C1	124.62 (8)
N1—Co1—O1	75.53 (3)	C2—C3—C4	117.64 (8)
N2—Co1—O1	94.87 (3)	C2—C3—H3A	121.2
O3—Co1—O1	94.97 (3)	C4—C3—H3A	121.2
O2—Co1—O1	151.77 (3)	C3—C4—C5	120.09 (9)
N1—Co1—O4	104.80 (3)	C3—C4—H4A	120.0
N2—Co1—O4	75.52 (3)	C5—C4—H4A	120.0
O3—Co1—O4	151.74 (3)	C6—C5—C4	118.54 (9)
O2—Co1—O4	98.20 (3)	C6—C5—H5A	120.7
O1—Co1—O4	87.21 (3)	C4—C5—H5A	120.7
O3W—Co2—O3W^i^	180.0	N1—C6—C5	120.72 (8)
O3W—Co2—O2W^i^	91.06 (3)	N1—C6—C7	113.36 (8)
O3W^i^—Co2—O2W^i^	88.94 (3)	C5—C6—C7	125.90 (8)
O3W—Co2—O2W	88.94 (3)	O6—C7—O2	126.75 (8)
O3W^i^—Co2—O2W	91.06 (3)	O6—C7—C6	118.39 (8)
O2W^i^—Co2—O2W	180.0	O2—C7—C6	114.83 (8)
O3W—Co2—N3^i^	89.13 (3)	O7—C8—O3	127.12 (9)
O3W^i^—Co2—N3^i^	90.87 (3)	O7—C8—C9	117.84 (8)
O2W^i^—Co2—N3^i^	88.54 (3)	O3—C8—C9	115.04 (8)
O2W—Co2—N3^i^	91.46 (3)	N2—C9—C10	121.34 (8)
O3W—Co2—N3	90.87 (3)	N2—C9—C8	113.16 (8)
O3W^i^—Co2—N3	89.13 (3)	C10—C9—C8	125.47 (8)
O2W^i^—Co2—N3	91.46 (3)	C9—C10—C11	118.39 (8)
O2W—Co2—N3	88.54 (3)	C9—C10—H10A	120.8
N3^i^—Co2—N3	180.0	C11—C10—H10A	120.8
C1—O1—Co1	116.61 (6)	C10—C11—C12	119.95 (8)
C7—O2—Co1	115.83 (6)	C10—C11—H11A	120.0
C8—O3—Co1	116.32 (6)	C12—C11—H11A	120.0
C14—O4—Co1	114.34 (6)	C13—C12—C11	117.80 (8)
C6—N1—C2	121.15 (8)	C13—C12—H12A	121.1
C6—N1—Co1	119.12 (6)	C11—C12—H12A	121.1
C2—N1—Co1	119.68 (6)	N2—C13—C12	121.93 (8)
C13—N2—C9	120.58 (8)	N2—C13—C14	113.57 (7)
C13—N2—Co1	120.38 (6)	C12—C13—C14	124.50 (8)
C9—N2—Co1	118.95 (6)	O8—C14—O4	126.69 (8)
C16—N3—N4	103.98 (7)	O8—C14—C13	117.20 (8)
C16—N3—Co2	135.05 (6)	O4—C14—C13	116.11 (8)
N4—N3—Co2	116.28 (5)	N4—C15—N6	124.95 (9)
C15—N4—N3	110.73 (7)	N4—C15—N5	107.43 (8)
C15—N4—H1N4	124.9 (11)	N6—C15—N5	127.62 (8)
N3—N4—H1N4	117.3 (11)	N3—C16—N7	125.34 (8)
C15—N5—C16	106.34 (7)	N3—C16—N5	111.49 (8)
C15—N5—H1N5	127.1 (13)	N7—C16—N5	123.16 (8)
C16—N5—H1N5	126.4 (13)	H1W1—O1W—H2W1	104 (2)
C15—N6—H1N6	117.0 (12)	Co2—O2W—H1W2	115.8 (13)
C15—N6—H2N6	121.6 (12)	Co2—O2W—H2W2	115.0 (14)
H1N6—N6—H2N6	121.3 (17)	H1W2—O2W—H2W2	107.6 (18)
C16—N7—H1N7	117.6 (13)	Co2—O3W—H1W3	122.4 (14)
C16—N7—H2N7	124.3 (13)	Co2—O3W—H2W3	115.8 (13)
H1N7—N7—H2N7	118.2 (18)	H1W3—O3W—H2W3	104.9 (18)
			
C16—N3—N4—C15	−0.82 (10)	C13—N2—C9—C8	177.27 (8)
Co2—N3—N4—C15	−160.26 (6)	Co1—N2—C9—C8	0.78 (10)
Co1—O1—C1—O5	−171.62 (7)	O7—C8—C9—N2	−176.02 (9)
Co1—O1—C1—C2	7.05 (10)	O3—C8—C9—N2	3.74 (12)
C6—N1—C2—C3	−0.26 (14)	O7—C8—C9—C10	2.07 (14)
Co1—N1—C2—C3	−177.65 (7)	O3—C8—C9—C10	−178.16 (9)
C6—N1—C2—C1	−179.43 (8)	N2—C9—C10—C11	0.15 (14)
Co1—N1—C2—C1	3.18 (10)	C8—C9—C10—C11	−177.80 (9)
O5—C1—C2—N1	172.01 (8)	C9—C10—C11—C12	0.38 (14)
O1—C1—C2—N1	−6.75 (12)	C10—C11—C12—C13	−0.15 (14)
O5—C1—C2—C3	−7.13 (14)	C9—N2—C13—C12	1.16 (13)
O1—C1—C2—C3	174.11 (9)	Co1—N2—C13—C12	177.60 (7)
N1—C2—C3—C4	1.55 (15)	C9—N2—C13—C14	−178.31 (8)
C1—C2—C3—C4	−179.38 (9)	Co1—N2—C13—C14	−1.88 (10)
C2—C3—C4—C5	−1.50 (16)	C11—C12—C13—N2	−0.62 (14)
C3—C4—C5—C6	0.24 (16)	C11—C12—C13—C14	178.80 (9)
C2—N1—C6—C5	−1.10 (14)	Co1—O4—C14—O8	177.20 (8)
Co1—N1—C6—C5	176.31 (7)	Co1—O4—C14—C13	−2.90 (10)
C2—N1—C6—C7	−179.62 (8)	N2—C13—C14—O8	−176.90 (8)
Co1—N1—C6—C7	−2.21 (10)	C12—C13—C14—O8	3.64 (14)
C4—C5—C6—N1	1.09 (15)	N2—C13—C14—O4	3.20 (12)
C4—C5—C6—C7	179.41 (9)	C12—C13—C14—O4	−176.26 (9)
Co1—O2—C7—O6	−170.41 (8)	N3—N4—C15—N6	−178.78 (9)
Co1—O2—C7—C6	7.75 (10)	N3—N4—C15—N5	1.67 (10)
N1—C6—C7—O6	174.41 (8)	C16—N5—C15—N4	−1.82 (10)
C5—C6—C7—O6	−4.03 (14)	C16—N5—C15—N6	178.65 (9)
N1—C6—C7—O2	−3.91 (11)	N4—N3—C16—N7	178.47 (9)
C5—C6—C7—O2	177.65 (9)	Co2—N3—C16—N7	−27.99 (15)
Co1—O3—C8—O7	173.50 (9)	N4—N3—C16—N5	−0.36 (10)
Co1—O3—C8—C9	−6.24 (10)	Co2—N3—C16—N5	153.18 (7)
C13—N2—C9—C10	−0.92 (13)	C15—N5—C16—N3	1.37 (10)
Co1—N2—C9—C10	−177.40 (7)	C15—N5—C16—N7	−177.50 (9)

Symmetry code: (i) −*x*, −*y*, −*z*.

### Hydrogen-bond geometry (Å, º)

**Table:**

*D*—H···*A*	*D*—H	H···*A*	*D*···*A*	*D*—H···*A*
N4—H1*N*4···O1^ii^	0.88 (2)	1.74 (2)	2.6044 (12)	165 (2)
N5—H1*N*5···O7^iii^	0.89 (2)	1.77 (2)	2.6402 (12)	169 (2)
N6—H1*N*6···O2	0.84 (2)	2.15 (2)	2.9117 (12)	151.1 (19)
N6—H2*N*6···O1*W*^iii^	0.92 (2)	1.85 (2)	2.7645 (12)	173.6 (19)
N7—H2*N*7···O6^iv^	0.80 (2)	2.24 (2)	2.9956 (13)	158.4 (18)
O1*W*—H1*W*1···O3	0.83 (2)	1.90 (2)	2.7354 (13)	171 (3)
O1*W*—H2*W*1···O6^v^	0.79 (2)	2.09 (2)	2.8711 (12)	175 (2)
O2*W*—H1*W*2···O5^ii^	0.86 (2)	1.80 (2)	2.6666 (11)	173 (2)
O2*W*—H2*W*2···O4	0.82 (2)	2.00 (2)	2.8206 (11)	175 (2)
O3*W*—H1*W*3···O4^ii^	0.79 (2)	2.19 (2)	2.9602 (11)	163 (2)
O3*W*—H2*W*3···O8^i^	0.83 (2)	1.76 (2)	2.5795 (11)	173 (2)
C3—H3*A*···O5^vi^	0.95	2.25	3.1816 (13)	167
C5—H5*A*···N7^vii^	0.95	2.50	3.4265 (15)	164
C10—H10*A*···O6^iii^	0.95	2.44	3.3898 (13)	177

Symmetry codes: (i) −*x*, −*y*, −*z*; (ii) *x*−1, *y*, *z*; (iii) −*x*+1, *y*−1/2, −*z*+1/2; (iv) *x*, *y*−1, *z*; (v) *x*+1, *y*, *z*; (vi) −*x*+2, −*y*+1, −*z*; (vii) *x*, *y*+1, *z*.
