# Supplementary figures and images for: Crystal structure of tetra­aqua­bis(3,5-di­amino-4H-1,2,4-triazol-1-ium)cobalt(II) bis­[bis­(pyridine-2,6-di­carboxyl­ato)cobaltate(II)] dihydrate
Source: Acta Crystallogr E Crystallogr Commun. 2015 May 30;71(Pt 6):m139–40. doi: 10.1107/S2056989015010014 (PMC4459343; doi:10.1107/S2056989015010014)

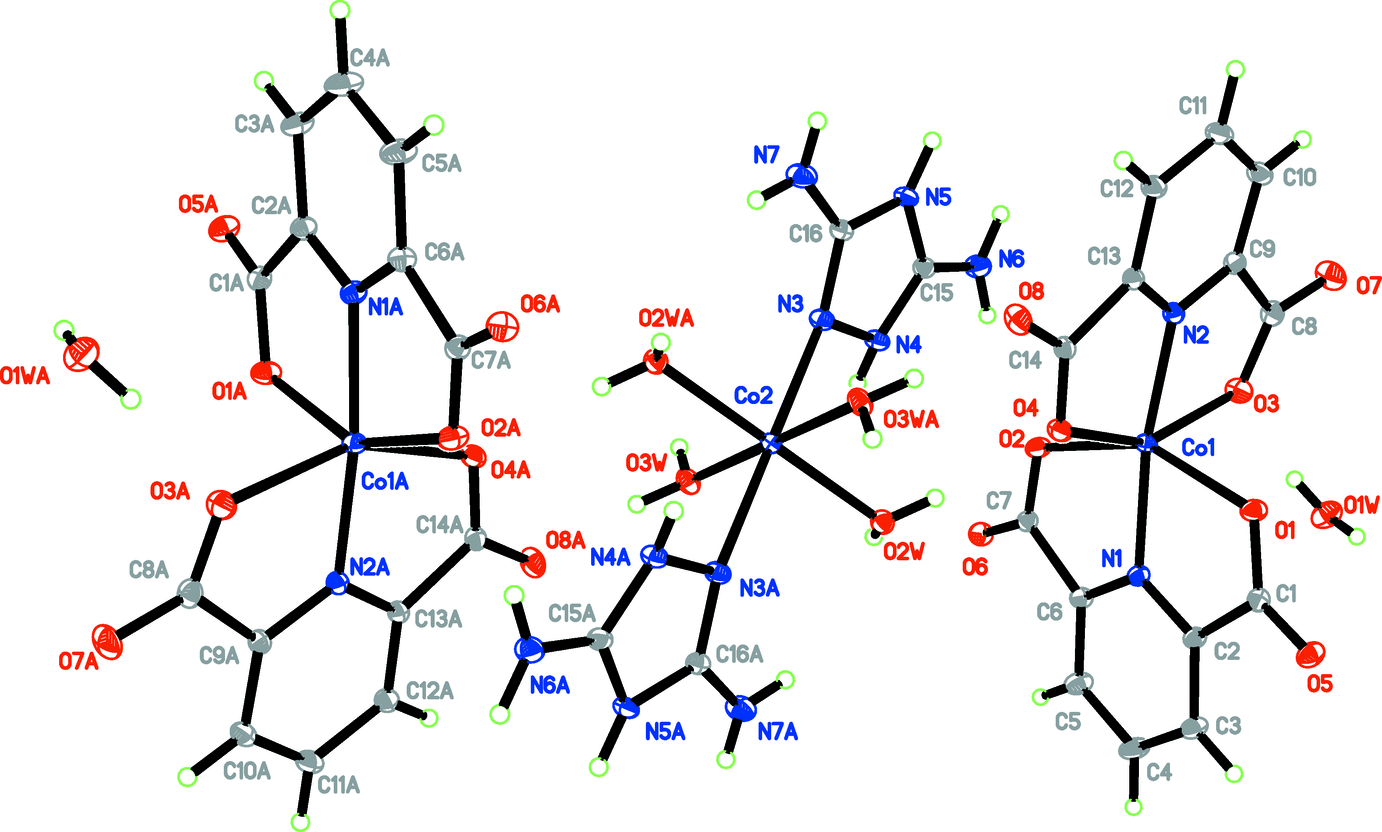

Supplement: Supplementary file 3 [file e-71-0m139-fig1.tif]

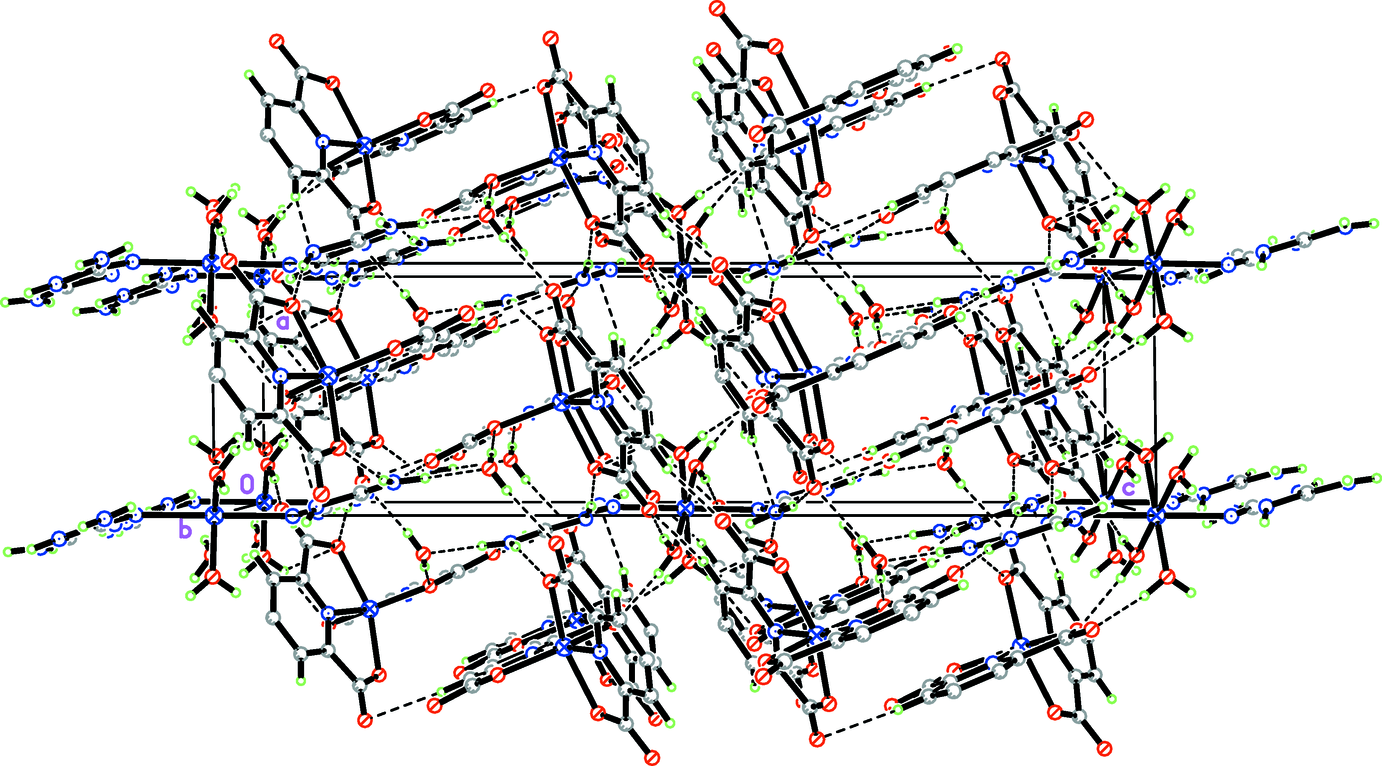

Supplement: Supplementary file 4 [file e-71-0m139-fig2.tif]
